# Quality assurance for intraoperative MRI RF coils in stereotactic neurosurgical planning

**DOI:** 10.1002/acm2.70699

**Published:** 2026-07-15

**Authors:** James C. Thorpe, Rafal Panek, Charlotte Bull, Paul S. Morgan

**Affiliations:** ^1^ Medical Physics Nottingham University Hospitals NHS Trust Nottingham UK; ^2^ School of Medicine University Of Nottingham Nottingham UK

**Keywords:** decision making, imaging, MRI, neurosurgery, quality assurance

## Abstract

**Background:**

The application of MRI during a surgical procedure (intraoperative MRI) provides the surgical team with up‐to‐date anatomical information to improve precision and inform decision making during the operation. This requires the use of a stereotactic frame or RF coil that affixes to the patient throughout the procedure, with the clinical team requiring assurance that the image quality is consistent and appropriate. The following QA program has been developed in order to monitor the condition of an intraoperative head coil.

**Methods:**

This was set up for a third party intraoperative 8‐channel head coil in conjunction with a 3.0T MRI scanner. QA testing consists of acquiring a large Field of View isometric (1.5 mm) 3D T1‐weighted rapid gradient echo (FFE) of a 5000 CC Phantom Bottle, with two dynamic acquisitions and the individual channel data saved. Offline analysis is then performed to calculate the signal‐to‐noise ratio (SNR) of the phantom within the coil, the SNR for each channel, and the distance (D) from the coil at which the SNR decreases to half the value within the coil.

**Results:**

Testing has been performed on a monthly basis since May 2022 with consistent values for SNR (including for each channel) and D. There were three occasions where the coil failed the QA tests.

**Conclusion:**

A monthly QA program has been developed to monitor coil element performance, confirming that the coil has been functioning as expected.

## 1 INTRODUCTION

Acquiring MRI during brain tumor surgery, intraoperative MRI, provides updated anatomical information following brain shift relative to pre‐operative anatomical images. This allows for the immediate assessment of the extent of tumor resection, localization of residual tumor, and rapidly identifies potential surgical complications such as intracranial hemorrhage.^1^, ^2^ Performing an intraoperative MRI therefore improves the potential for safe tumor resection, reduces the chance of early reoperation, and improves overall survival^3^, ^4^ in a range of clinical applications.^5^

Intraoperative MRI guided tumor resection requires the patients head to be affixed to a stereotactic frame for the duration of the procedure. In order for images to be acquired, the stereotactic frame must either have an inbuilt radiofrequency (RF) coil or else an additional RF coil must be used in conjunction with the frame at the point of scanning.^6^, ^7^ The frame must also contain a specific configuration of fiducial markers that are visible in MRI and to cameras in the operating theatre to allow stereotactic planning.

Neuro‐stereotactic procedures are reliant on high levels of accuracy and precision in order to be safe and effective with several publications reporting accuracies of the order of one millimeter.^8^, ^9^, ^10^ However, MR image distortion and image quality issue can arise from metal susceptibility issues from pins and other metal in the frame, radiofrequency noise from inadequately shielded electronics in the operating room, the interface between the brain and introduced air from the operation, and blood products.^11^ Furthermore, patient positioning affects brain position, with brain shift during an operation measured with MRI as 1–24 mm, depending on depth.^12^ The importance of high levels of accuracy combined with many factors potentially increasing targeting error necessitates robust and relevant quality control for intraoperative MRI planning scans.

Standard diagnostic coils usually have defined Quality Assurance (QA) procedures designed and established by the manufacturer. These are typically established following the American Association of Physics in Medicine (AAPM) Guidelines and American College of Radiology (ACR) accreditation.^13^, ^14^ This stipulates the importance of testing RF coils, including the assessment of individual coil elements for standard diagnostic coil arrangements. However, the coil configurations used for intraoperative MR acquisition are not covered by this guidance. Typically, manufactures therefore do not provide specific QA procedures for the configurations used in intraoperative MRI.

Clinical MRI QA programs are greatly concerned with monitoring SNR, which is sensitive to detecting changes of system specific characteristics^15^ and also the receiver coil used for the test. Tolerances are typically provided reflecting SNR levels expected for a coil in a good working condition providing adequate signal in clinical scans.

RF coil performance is of increased importance intraoperatively relative to standard diagnostic MRI as the patient is affixed to the coil for the duration of the procedure. Additionally, in the intraoperative MRI setting the area of clinical interest can potentially be outside the central region of the RF coil due to surgical considerations in pinning preventing the coil from covering the entire head. It is therefore desirable that the image quality at a distance from the coil is monitored and maintained. It is also the case that intraoperative MRI requires assurance not only of the image quality but also the ability to identify the fiducials.

The QA program described here has been developed in order to monitor the condition of a third party intraoperative head coil independent of any existing QA program established for the MRI scanner and standard RF coils.^15^

## 2 METHODS

**FIGURE 1:**
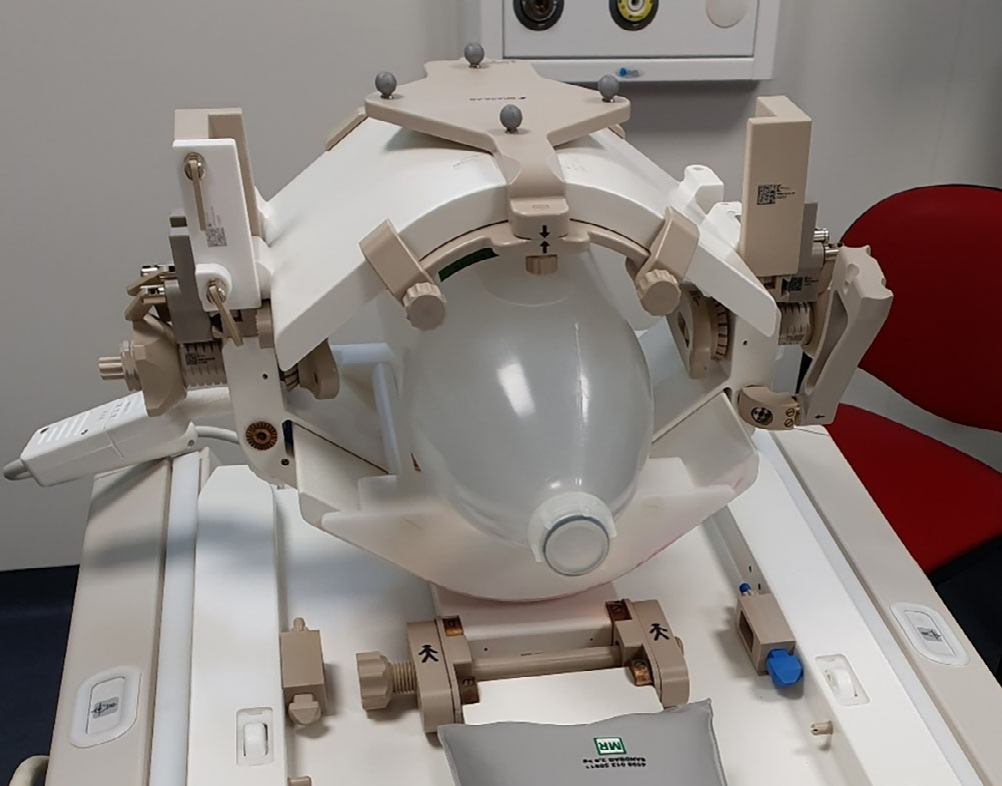
Phantom bottle and coil setup for QA testing.

The QA program was set up for a NORAS MRI Products intraoperative 8‐channel head coil (Noras MRI products, Höchberg, Germany) and performed on a monthly basis using a Philips Ingenia Elition X 3.0T MRI scanner (Philips Medical, Best, The Netherlands). This combination of scanner and RF coil is routinely used for intraoperative MRI. The program began in May 2022 and has been operating since then. Image quality is assessed by using a standard Philips 5000 CC (cubic centimeter) Phantom Bottle (Contents: Spectrasyn 4 P/N 4598 000 51611) with a Philips phantom holder used to facilitate reproducible positioning within the coil as shown in Figure 1 (full detail on the setup process is given in the Supplementary Material).

The imaging protocol consists of a localizer scan followed by a 1.5 mm isometric 3D T1‐weighted rapid gradient echo (FFE) with two dynamics (repetition time = 5.8 ms, echo time = 2.6 ms, flip angle = 8°, NSA = 1, BW = 285 Hz/pixel, total acquisition time = 06:59) with a large field of view (FOV) (400 × 322 × 250 mm). No parallel imaging is used. Coverage includes the whole phantom and fiducial markers which are located on the interior surface of the anterior coil. The image data from each of the eight individual channels of the RF coil are also saved. The full image data and individual channel images are then sent to a workstation for analysis. The full QA image acquisition protocol is given in the supplementary information.

**FIGURE 2:**
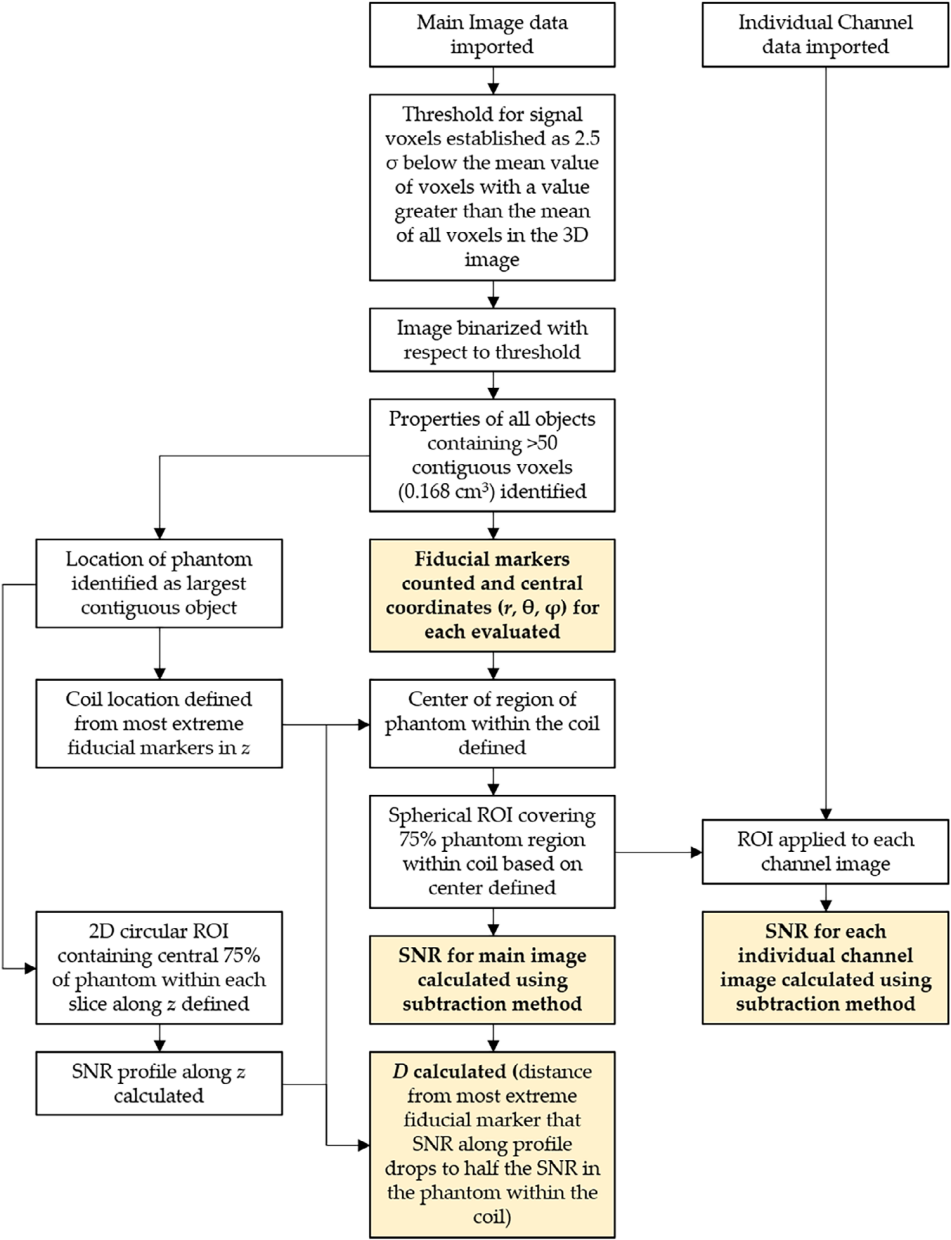
Block diagram of offline analysis with steps producing output metrics highlighted.

Offline analysis, shown as a block diagram in Figure 2, is performed by identifying the positions of the 5000 CC Phantom Bottle and 14 fiducial markers (located on the interior of the anterior component of the coil) within the image (Matlab, MathWorks). This is done by identifying the mean value of all voxels before repeating this to identify the mean value and standard deviation of all pixels with a value greater than the overall mean. A threshold of 2.5 standard deviations below the mean of these pixels is established as the limit of which pixels contain signal. Any pixel with a value below this threshold is considered to only contain noise. The image is then binarized with all voxels containing only noise given a value of zero. All groups of contiguous signal voxels could then be readily assessed in terms of total number of voxels per object and object position.

The number of fiducials identified by the analysis is verified to ensure all are present. The relative coordinates of the center of the fiducial markers in relation to the center of the phantom is evaluated as a test of consistency and equipment setup. These are reported in terms of distance (r), azimuth angle (θ) and polar angle (φ) to the center of all fiducials. The locations of the two extreme fiducials in the z direction are taken as reference points for where the coil is located. This is needed as the phantom extends outside of the coil with the fiducial markers being the only component of the coil visible in the MR image.

A spherical region of interest (ROI) is then defined covering 75% of the volume of the phantom contained within the two most extremely located fiducial markers. This is done by identifying the center and extent of the volume of the phantom between the two extreme markers. A spherical ROI is then defined within this region with a radius such that that ROI covered 75% of this phantom volume.

The signal‐to‐noise ratio (SNR) within this region is then calculated using the NEMA subtraction method.^16^ This is done by taking two images acquired immediately after one another such that the only difference between the images is the noise. One of these two images is defined as being the main “image” containing the signal. The other image is then subtracted from main image in order to produce a subtraction image containing only noise. The same ROI is then applied to both images and the mean signal of the image ROI and standard deviation of the subtraction image ROI is evaluated. The SNR can then be calculated using Equation 1:

(1) $$\mathrm{SNR}\;=\sqrt{2}\;\frac{\mathrm{mean}\;\mathrm{signal}\;\mathrm{in}\;\mathrm{image}\;\mathrm{ROI}}{\mathrm{standard}\;\mathrm{deviation}\;\mathrm{in}\;\mathrm{subtraction}\;\mathrm{image}\;\mathrm{ROI}}$$

where the factor of $\sqrt{2}$ is needed to correct for the increased standard deviation caused by the subtraction image containing noise from both acquired images.^16^ The same ROI is then selected in each individual coil element image and the SNR within this ROI calculated in the same way.

**FIGURE 3:**
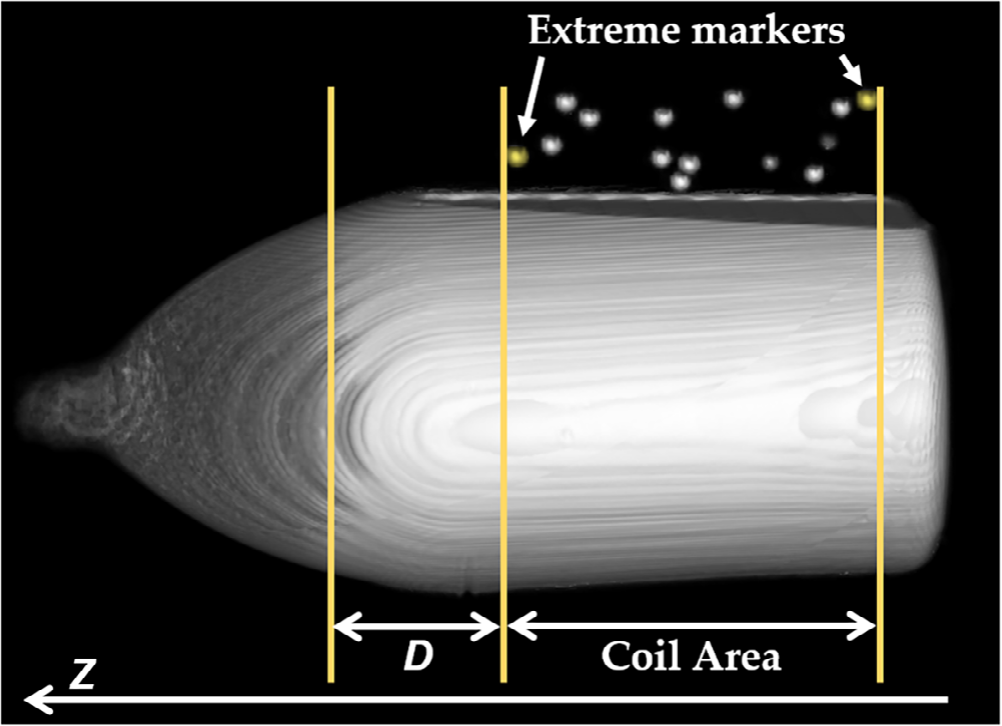
3D rendering of phantom and fiducial markers highlighting the extreme fiducial markers, the area known to be within the coil and the measured distance, D.

Signal drop‐off outside of the coil is assessed by calculating a single line SNR profile positioned centrally along the length of the bottle. This is done by identifying the center of the phantom within each slice of the image, and defining a circular ROI with a radius such that 75% of the phantom within that slice is included. The SNR of the ROI in each slice is then calculated using the subtraction method. This is repeated for every slice in the z direction for the combined image. The SNR for each slice is then compared to the SNR calculated from the main ROI covering the phantom within the coil. The distance from the most extreme fiducial to the point the SNR along the profile drops to half the value of that within the coil, denoted “D”, is calculated, as shown in Figure 3.

The mean signal intensity for each of the fiducial markers are also calculated by defining an ROI in the central 75% of each fiducial. This was done by defining a spherical ROI centered on each fiducial with a radius such that 75% of the fiducial was included within the ROI. Signal intensity within each fiducial marker was assessed in order to identify any trends or deterioration over time. The signal of the fiducial markers was used as an assessment metric rather than SNR as the volume of the markers were less than 10 cubic voxels making the calculation of SNR unreliable. The number of voxels present in the fiducial markers is insufficient for the characterization of noise contributing to fluctuations in the signal.^17^ Measuring signal instead of SNR causes an increased amount of variability within the measurements. However, if one fiducial maker becomes defective then a continuous declining trend away from the other fiducial markers is expected and this can be monitored.

In order to establish standard errors for each of the acquired metrics, baseline measurements were acquired by performing five consecutive repeated measurements on the coil and phantom. Threshold values for the main SNR, *D* and each individual element SNR have been calculated from the entire data set as three standard deviations either side of the mean. Any value below this threshold constitutes a failure of QA testing. Normal distribution of parameters was tested using Shapiro‐Wilk test with 0.05 significance level.

## 3 RESULTS

**FIGURE 4:**
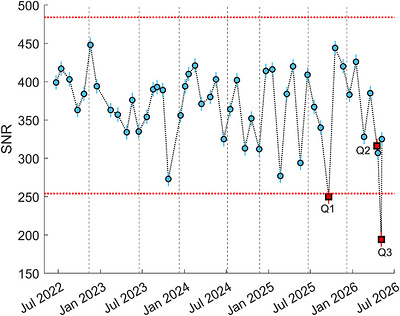
Signal‐to‐noise ratio (SNR) of bottle phantom in Noras Coil from June 2022 to May 2026 (*n* = 50) with thresholds (shown in red) defined as three standard deviations from the mean calculated from the complete data set (excluding test failures) plus an additional five measurements acquired for baseline error calculation. Dates of preventative maintenance are indicated with dashed gray lines. QA test failures indicated as Q1‐3.

Figure 4 shows the SNR of the phantom within the coil from May 2022 to May 2026 (*n* = 47, mean = 374, range = 273–448, coefficient of variation = 11.49%, values excluding three occasions on which QA testing failed). Although an initial steady downwards trend was apparent over the first year, this has stabilized in later tests (*R* ^2^ = 0.04). There have only been three occasions in which QA testing failed, two of which were because a measurement exceeded the lower threshold for SNR. These occasions are indicated in Figure 4 and labelled Q1, Q2 and Q3. On one occasion (Q1), images were interrogated, and no image quality or positioning issues were identified. The second failure (Q2) did not result in the main SNR threshold being exceeded but was instead identified by other metrics and determined to be due to the connector being improperly connected. The third occurrence (Q3) was due to the anterior component of the coil being incorrectly set to its maximum height (65 mm above the minimum). No further action was taken and there were no subsequent issues.

**FIGURE 5:**
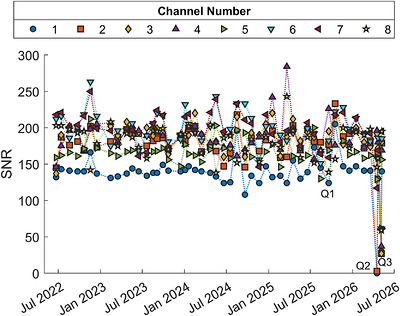
Signal‐to‐noise ratio (SNR) of bottle phantom in Noras Coil for each coil element from June 2022 to May 2026 (*n* = 50). QA test failures indicated as Q1‐3.

The SNR for each of the individual coil channels is shown in Figure 5 where again there are no visible trends in any channel with three occurrence of QA failure indicated. It can be seen that there have been no malfunctioning channels resulting in persistent signal dropout, however there were two occasions on which significant signal dropout can be seen. One of these was due to the coil being incorrectly connected (Q2), whilst the other issue came from improper setup with the anterior coil positioned too high (Q3). The occurrence of a borderline failure in main SNR (Q1) did not result in a failure in any other metric including individual channel SNR.

The distance (D) from the Noras Coil at which the SNR of the phantom drops to half the mean value of the phantom within the coil had a mean value of 73.5 mm with a standard deviation of 12.6 mm. There was no significant trend over time with this measurement (*R* ^2 ^= 0.01), however there is a moderate negative correlation between SNR and *D* (*R* ^2^ = 0.58, *p* < 0.001).

The distribution of SNR and *D* was normal (Shapiro‐Wilk *W *= 0.960 and *W *= 0.984 respectively, *p* > 0.05 for both).

The central location of all fiducial markers relative to the center of the phantom was tested and reported in terms of r, θ, and φ. The mean values of these locations across all measurements along with the range and coefficient of variation is given in Table 1.

**TABLE 1:** Summary of central location of fiducial markers relative to the center of the phantom reported in terms of distance (*r*), azimuth angle (θ) and polar angle (φ).

	Mean	Range	Coefficient of variation
*r*	139 mm	129–149 mm	2.86%
θ	1.93°	1.56–2.10°	5.57%
φ	−0.26°	−0.34 to −0.19°	−11.70%

The mean signal detected in the central region of each fiducial marker remained broadly consistent with a mean intensity for all fiducials of 763 and no observable trends or deterioration (excluding three failed tests). However, the signal in any given fiducial marker can fluctuate substantially with a mean signal range of 961 across all fiducials with the largest range being 1560. No atypical deviating trends (sudden consistent offsets or gradual trends up or down) were observed in any fiducial markers, however there was one occasion where the coil was not connected properly in which the signal in two fiducials dropped to the point that they were not locatable for analysis.

## 4 DISCUSSION

This QA program for testing stereotactic frames and RF coils for use in intraoperative MRI shows consistency in the metrics analyzed. There were three occasions in which QA testing failed in according to different metrics with the selection of failed metrics being critical in identifying the cause of the failure. One failure (Q2) would not have been identified if only the main SNR of the image were assessed. These incidents give confidence that this QA program can identify a range of potential issues with the coil.

It was not expected that the coil tested would show signs of deterioration in the time scale assessed and therefore this implies that the QA test is robust. Initially there was a strong downward trend in the main SNR measurement however this proved to be temporary and overall, the SNR has stayed within the expected thresholds (with one exception). The range of SNR values is therefore quite large, as gradient echo sequences are more variable than spin echo sequences.^17^ A gradient echo sequence was used over a single slice spin echo sequence to better assess the whole coil whilst keeping acquisition time down to a reasonable level (as no image acceleration used).

For the individual channel data, some channels have a consistently higher SNR than others. This is to be expected as the coil elements for the Noras coil are arranged in an oval around the coil. As a result, some elements are closer to the phantom than others and as such will have a higher SNR. It is important to monitor the ordering of the coil to ensure this is consistently reported. Software or coil changes can cause the ordering to change resulting in apparent fluctuations in the QA results.

The measurements of *D* indicate that the mean distance at which SNR decreases by half is on average 74 mm. If the tumor is further away from the coil than this distance then alternative coil configurations should be considered, and the overall benefit of attempting intraoperative MRI in the situation assessed.

The moderate negative correlation between *D* and SNR was unexpected as this suggests that the signal drop off is related to the signal detected. Further analysis of the SNR profile along the bottle is needed to better characterize this relationship. One possible cause of this potential relationship could be the effect of the scanner's uniformity correction on the image.

The fiducial markers were measured to be at a consistent position relative to the phantom, which implies a consistency in the testing set up with one exception (Q3) in which the anterior component was set to the highest height resulting in the QA test failing (with SNR for the main image and four channels dropping well below the threshold). We believe that this is an important test as the coil features many adjustable components including the height of the anterior component relative to the posterior component. A 20 mm range in the *r* metric was observed which can be attributed to the highly adjustable nature of the coil leading to slight variation in the exact height of the anterior component of the coil. The position of the anterior component of the coil varies for different clinical cases and so the variability in the position for the QA testing is not required to match the expected precision for stereotactic procedures. Further investigation could be performed to investigate the relative positions of each of the fiducial markers to longitudinally evaluate potential image distortion.

The required accuracy for stereotactic planning has been reported as being of the order of one millimeter.^8^, ^9^ However, there are various factors in an intraoperative setting that have the potential to increase targeting error including the frame, fixation pins, equipment around the scanning and operating rooms, introduced air, blood products, and patient position.^11^, ^18^ This QA program therefore gives neurosurgeons assurance that the RF coil is functioning correctly, increasing confidence that planning is accurate and minimizing risk of imaging failure during the procedure.

From experience of clinical cases of intraoperative MRI guided tumor resections at the site where this QA program was developed, it has been observed that this QA program provides reassurance during a case when technical issues arise. For example, in a case in which not all fiducial markers have been identified on a planning sequence, the QA program has given reason to assume all fiducials are still present and that the sequence needed to be replanned. This is less intuitive than it might initially appear as the fiducial markers are sometimes significantly above the patient's head particularly if the patient's head is small. Similarly poor image quality issues due to the coil not being connected properly have been rapidly recognized and resolved in part due to assurance that the coil is functioning properly because of this QA program.

Preliminary modifications suggest that this method would also be suitable for use with an alternative setup for intraoperative MRI cases. This includes using a DORO LUCENT cranial stabilization system in conjunction with Philips dStream Flex L coils. There are slight differences with this setup in that the frame only features 13 fiducial markers rather than the 14 in the Noras Coil, and the Flex L Coils only feature two channels rather than the eight for the Noras. This leads to a potential future multi‐site study with these tests being performed at another intraoperative MRI site using different coils.

On the basis of the findings of this program and our experiences, we suggest that intraoperative MRI services will find value in implementing this program especially given that QA is recommended in other specialist areas^15^ including MR treatment planning for radiotherapy^19^ or the NHS Breast Screening Program in the UK.^20^

## 5 CONCLUSION

A monthly QA program for the assessment of a third‐party intraoperative MRI coil has been developed in order to ensure accurate stereotactic planning. The results of this program confirm the coil has been functioning at expected SNR levels throughout its usage in intraoperative MRI cases. The functionality of each of the individual elements of the coil has also been validated, and the signal drop off from the coil established. There were three occasions in which the coil failed its QA testing for three different reasons captured in different sets of metrics indicating this program can identify a range of potential issues with the coil.

## AUTHOR CONTRIBUTIONS

**James C. Thorpe**: Conceptualization; methodology; validation; formal analysis; investigation; resources; data curation; writing – original draft preparation. **Rafal Panek**: Validation; writing – review and editing. **Charlotte Bull**: Data curation; writing – review and editing. **Paul S. Morgan**: Conceptualization; validation; writing – review and editing; supervision. All authors have read and agreed to the published version of the manuscript.

## CONFLICT OF INTEREST STATEMENT

The authors declare no conflicts of interest.

## Supporting information



Supporting Information
