# Supplementary material for: Quality assurance for intraoperative MRI RF coils in stereotactic neurosurgical planning
Source: J Appl Clin Med Phys. 2026 Jul 15;27(7):e70699. doi: 10.1002/acm2.70699 (PMC13373452; doi:10.1002/acm2.70699)
Supplement: Supplementary file 1 — Supporting Information [file ACM2-27-e70699-s001.docx]

# Supplementary Materials

| NORAS QA Image Acquisition |
| --- |

**1. PURPOSE**

To ensure that routine quality assurance checks are made on the 8ch NORAS Head coil used for intraoperative Magnetic Resonance Imaging (MRI) cases in an appropriate and consistent manner.

**2. SCOPE AND CONSTRAINTS**

This protocol can be performed by any MR Authorised Person with appropriate training in this protocol.

**3. EQUIPMENT NEEDED**

Image acquisition requires the following equipment:

1. 8 Channel NORAS Head Coil
2. 5L bottle
3. Phantom Holder
4. Y-Adapter Cable
5. Manual Table (instructions for use in Appendix A)

It is worth noting that the Head Coil can be disassembled into numerous parts that must all be present, however in typical practice the coil can be treated as one item as it is often stored in one piece.


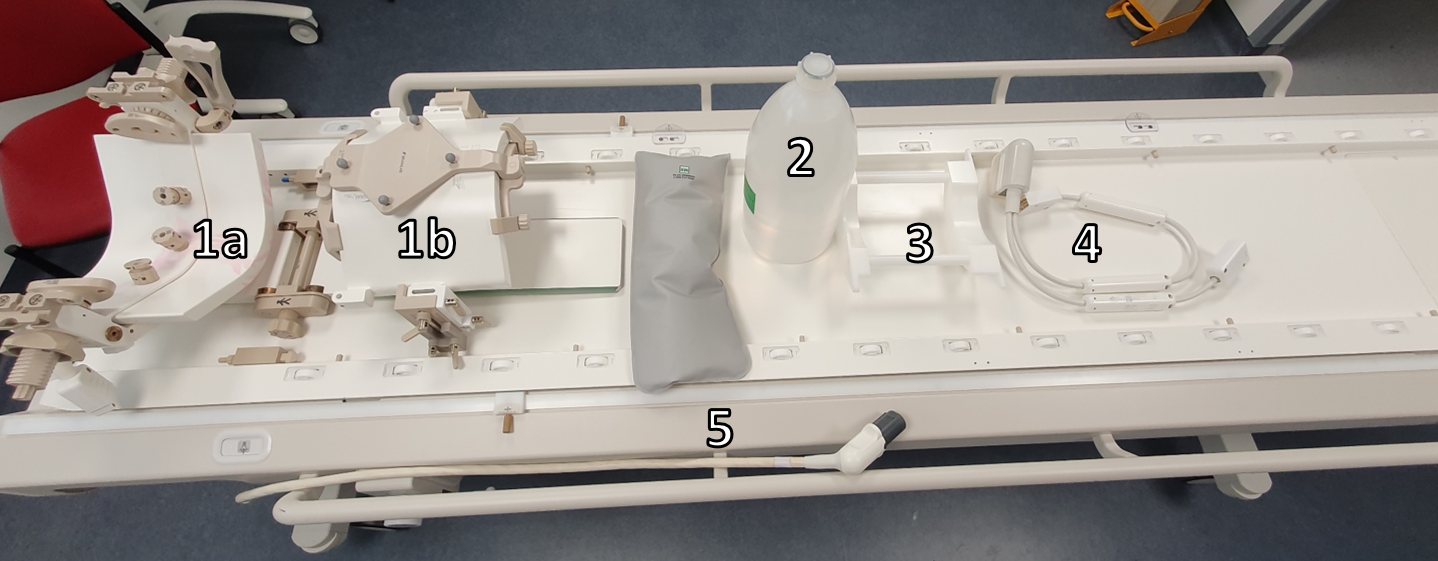


Figure 1 Equipment needed for QA Testing (NORAS coil split into posterior and anterior component parts [1a and 1b]).

**4. SETTING UP THE COIL & PHANTOM**

**Step 1 –** Perform a visual inspection of the coil to check for any visual damage. If any damage is seen, check in QA log folder to see if it is new and if so photograph and record in folder.

**Step 2** – Plug the Y-Adapter cable [4] into the port at head of the manual bed [5] on the short edge as shown in Figure 2.


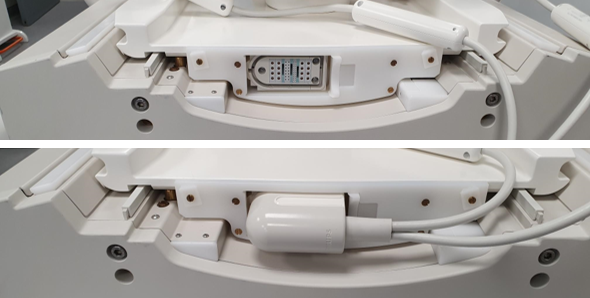


Figure 2 position of port for Y-adapter at head of bed

**Step 3** – If the coil has been delivered with the anterior coil attached to the posterior coil then remove the anterior coil. This is done by unscrewing the screws on both sides of the coil against the grey background indicated by red arrows in Figure 3. Note: the screws only need loosening until the coil can be detached; they do not need to be fully removed.


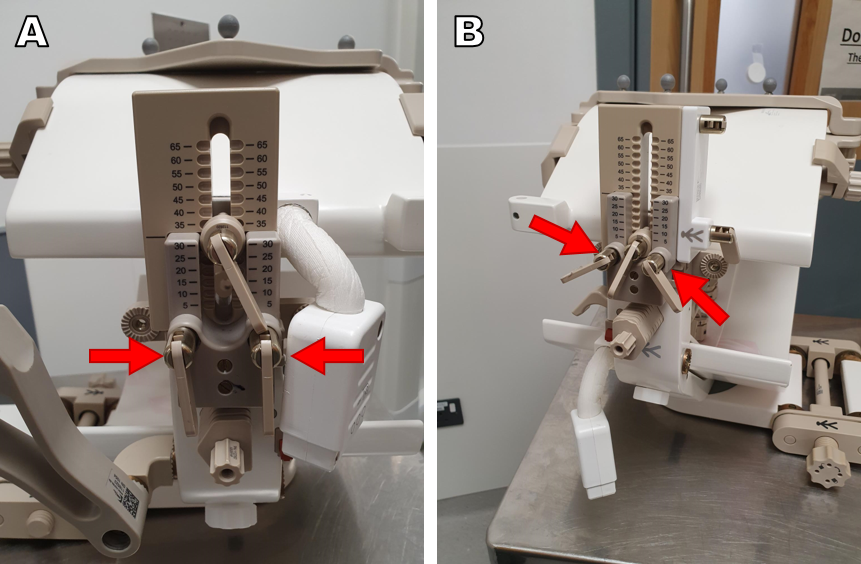


Figure 3 location of screws on both sides of coil (A & B) for removing and attaching anterior part of coil [1b] indicated with red arrows

**Step 4 –** Place the posterior coil at the head end of the table as shown in Figure 4.

**
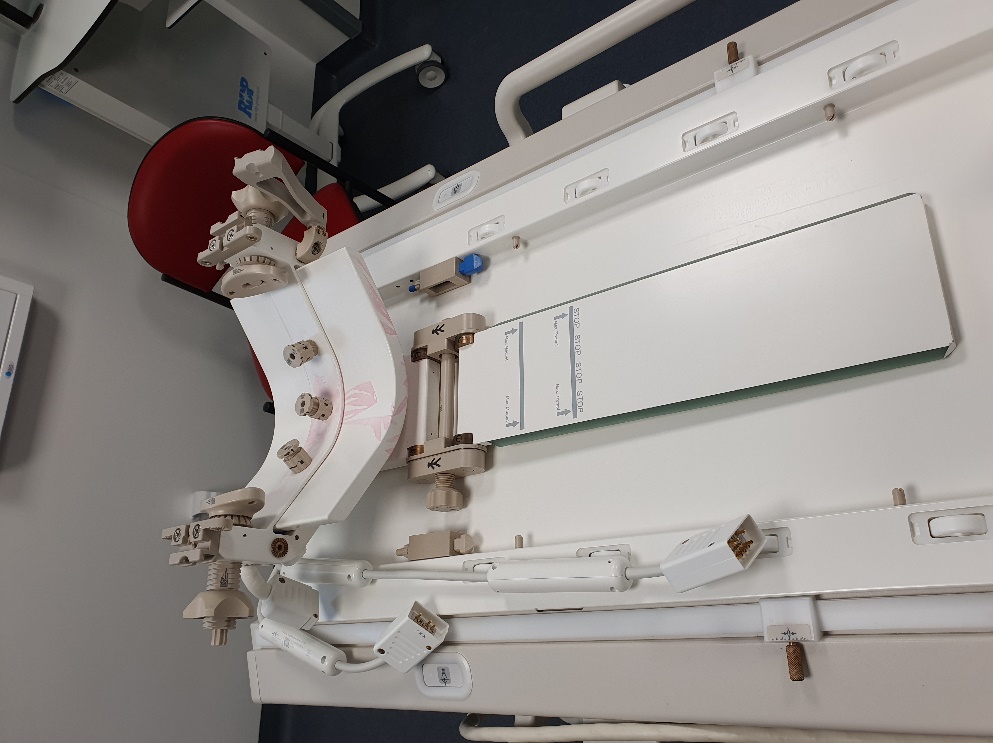
**

Figure 4 posterior NORAS coil in correct position

**Step 5** – Remove the three pin holder screws in middle of posterior component of head coil [1a] and place to one side.


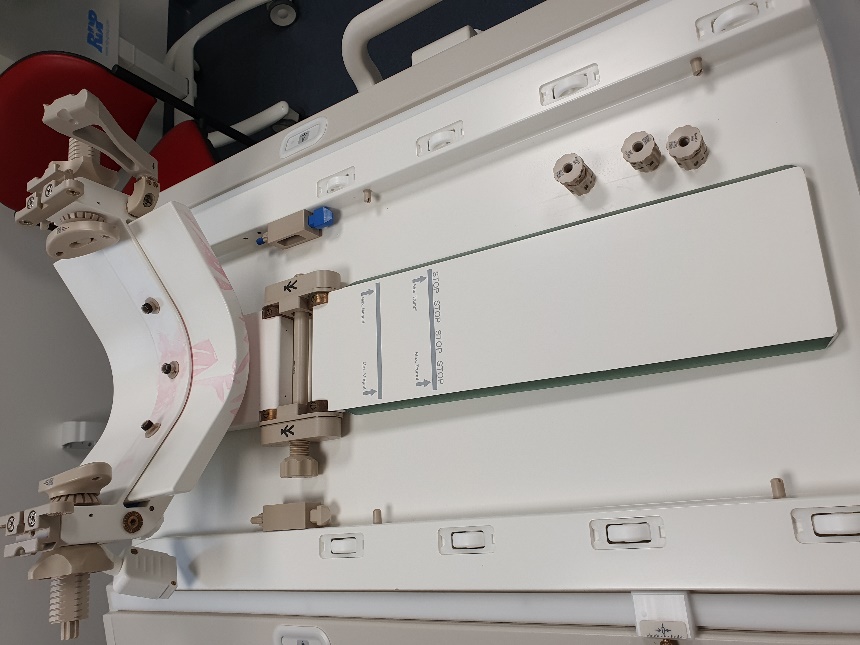


Figure 5 pin holder screws removed

**Step 6** – Place the phantom holder [3] on the posterior head coil [1a].


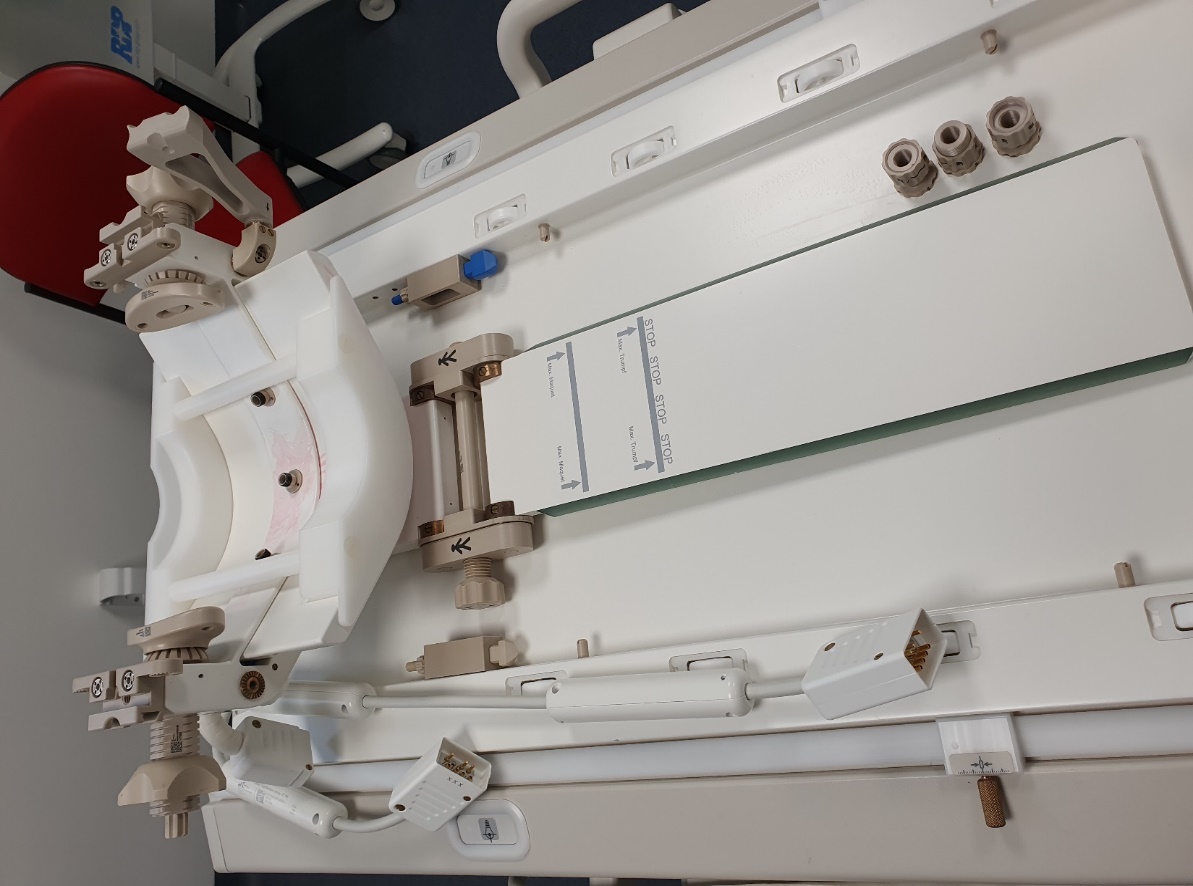


Figure 6 phantom holder positioned

**Step 7** – Place the 5L bottle phantom [2] in the phantom holder [3]. Note: it is advisable to place a sandbag on the other end of the coil to stop it from tipping.


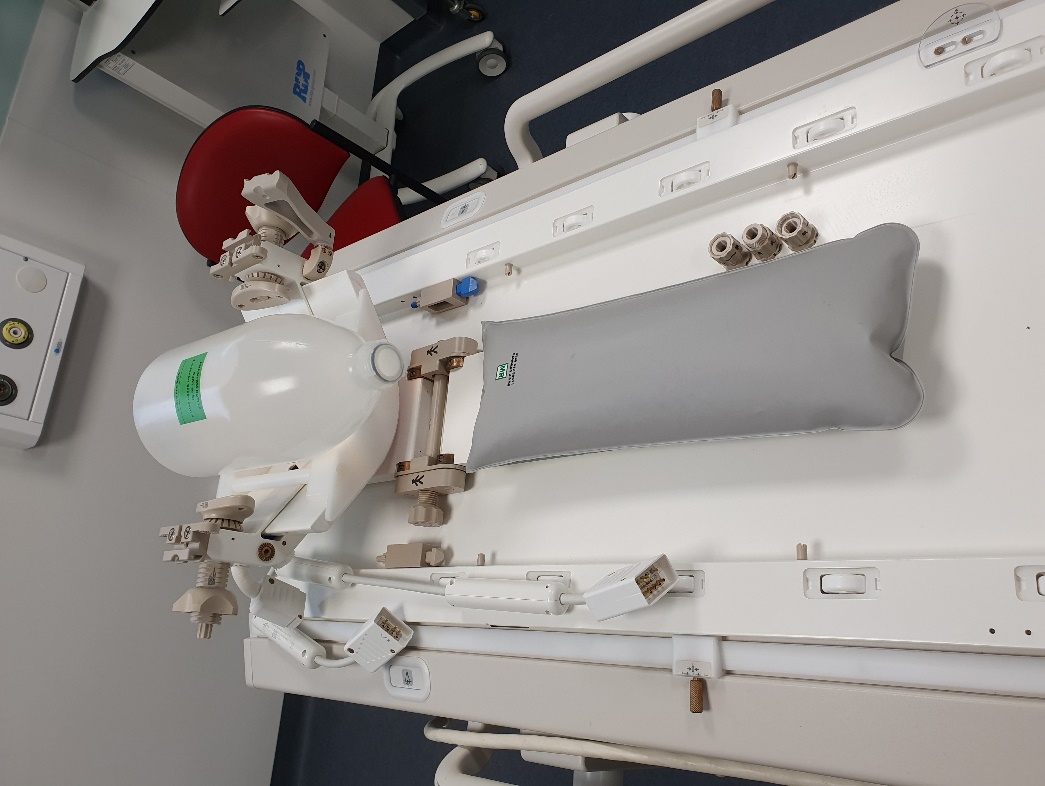


Figure 7 5L bottle phantom in position

**Step 8** – Attach anterior part of coil [1b] to posterior part of NORAS coil [1a] using the screws in the outer grey section on each side (shown in Figure 3 with red arrows), and plug in Y-adaptor [4].


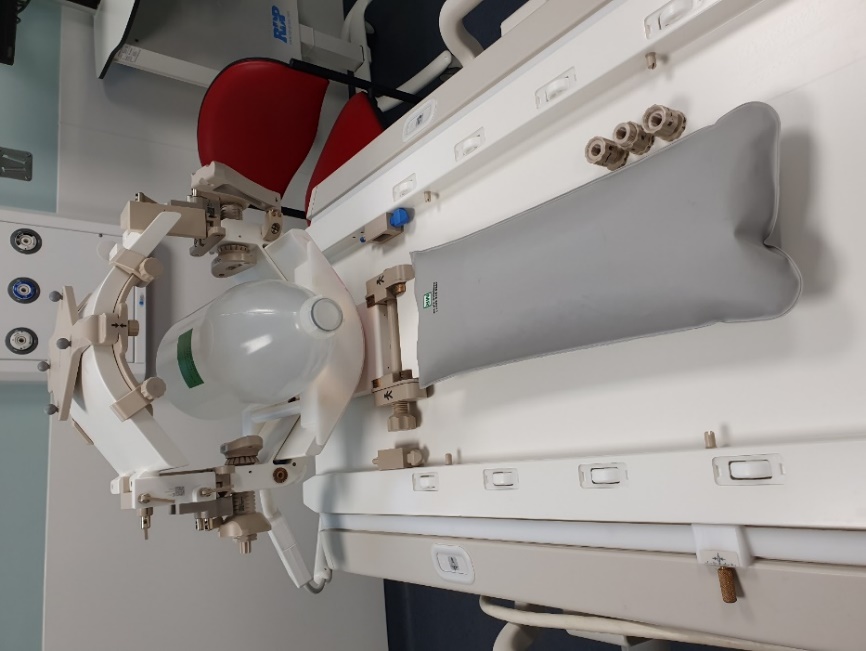


Figure 8 Anterior component of coil added

**Step 9** – Set the anterior part of the coil [1b] to the lowest height using the innermost screws (one on each side in the beige section) bringing it as close as possible to the phantom [2]. The relevant screws are shown in Figure 9 including two additional screws on one side on a white backdrop, which may also need to be loosened in order to allow the coil height to be adjusted (all four screws indicated with red arrows).


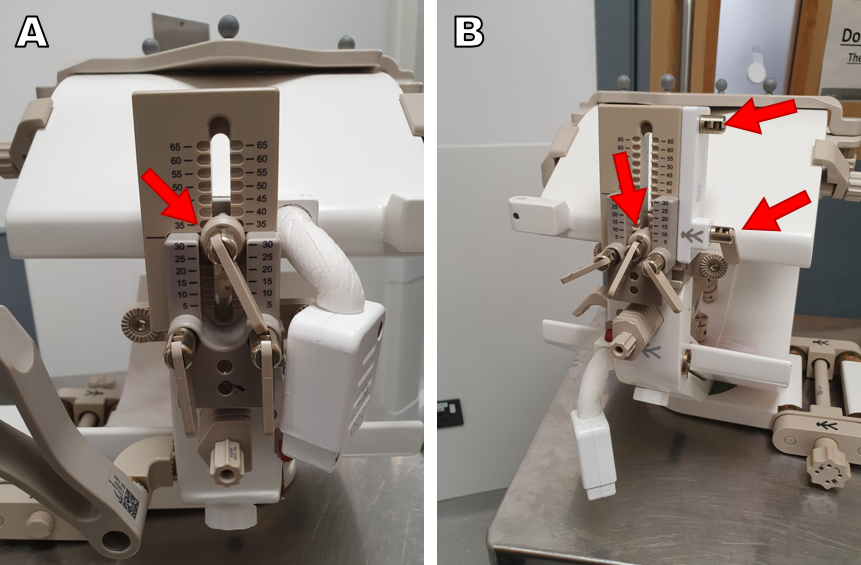


Figure 9 location of screws needed to adjust coil height


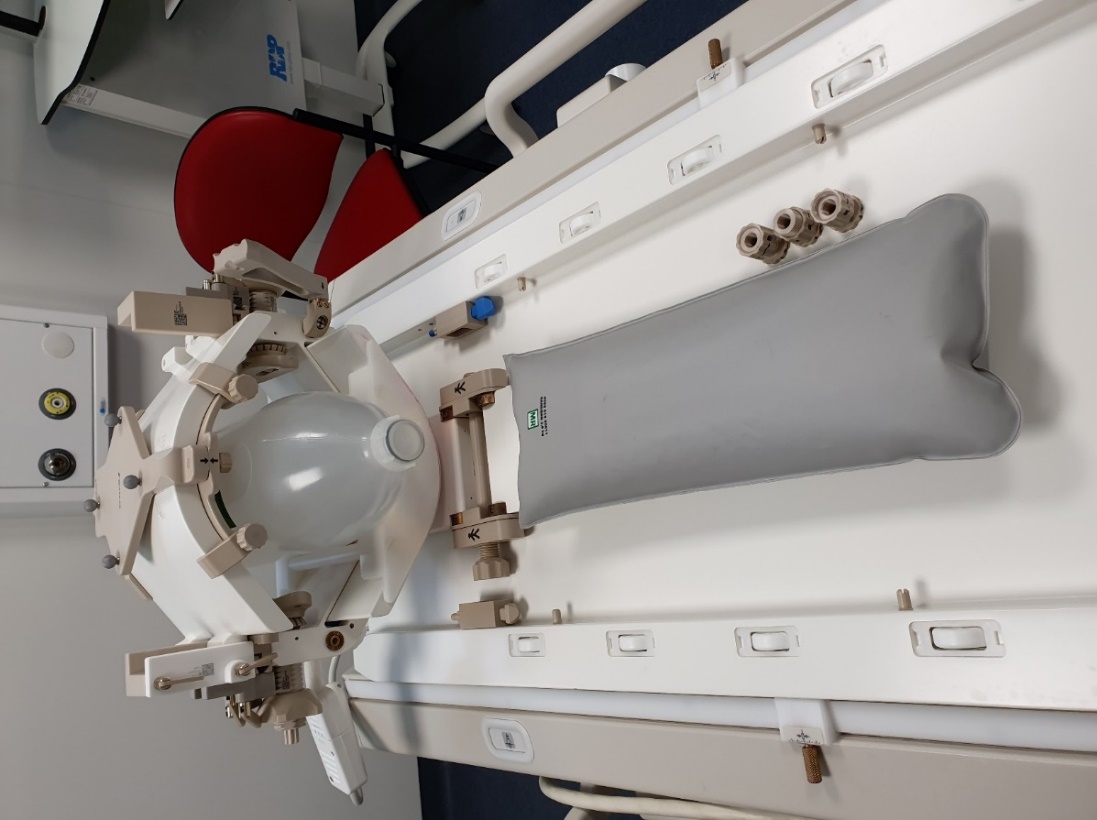


Figure 10 coil and phantom set up

**Step 10** – Plug coil [1a & 1b] into Y-adapter [4] such that the two leads marked “XXX” are connected on one side, and the two leads marked “OOO” are connected on the other side, as shown in Figure 11. Note: the lead connectors push together with no locking mechanism.


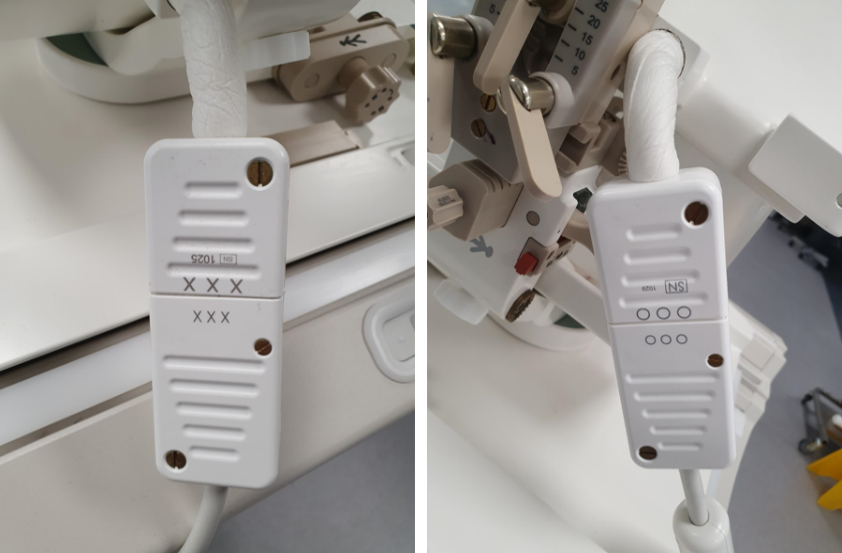


Figure 11 Coil leads plugged into correct Y-adapter leads

**4. POSITIONING THE COIL**

Once the phantom is positioned the coil should be aligned with the laser such that the crosshairs go through the middle of the “B”s and “I” in the word “BRAINLAB” written on top of the coil as shown in Figure 12.


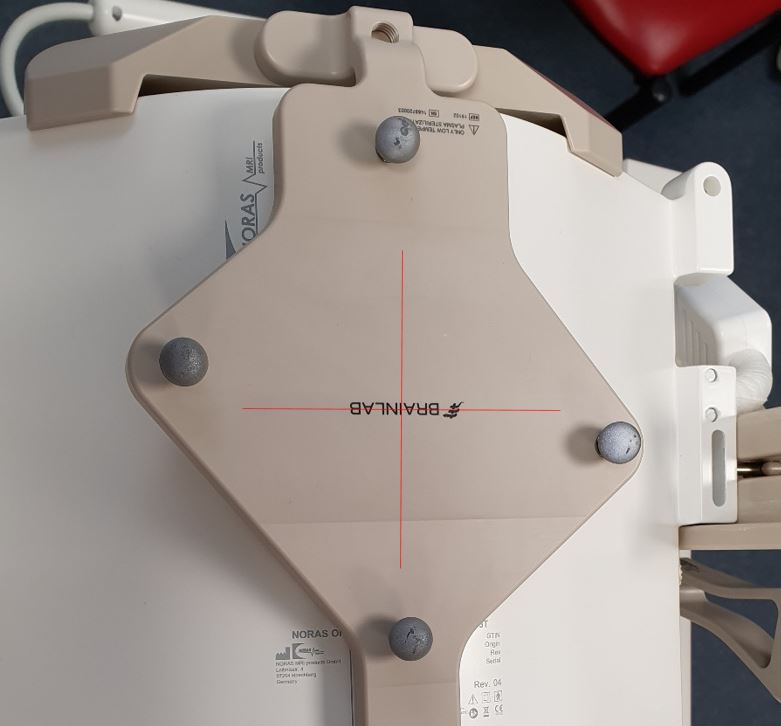


Figure 12 correct alignment of laser using word "BRAINLAB" as a guide

One side of the manual bed features two clear plastic markers (note these markers are not the coil fiducial markers and are independent of the coil) and a brass screw shown in Figure 13 – markers highlighted by red circles, screw indicated with a red arrow.


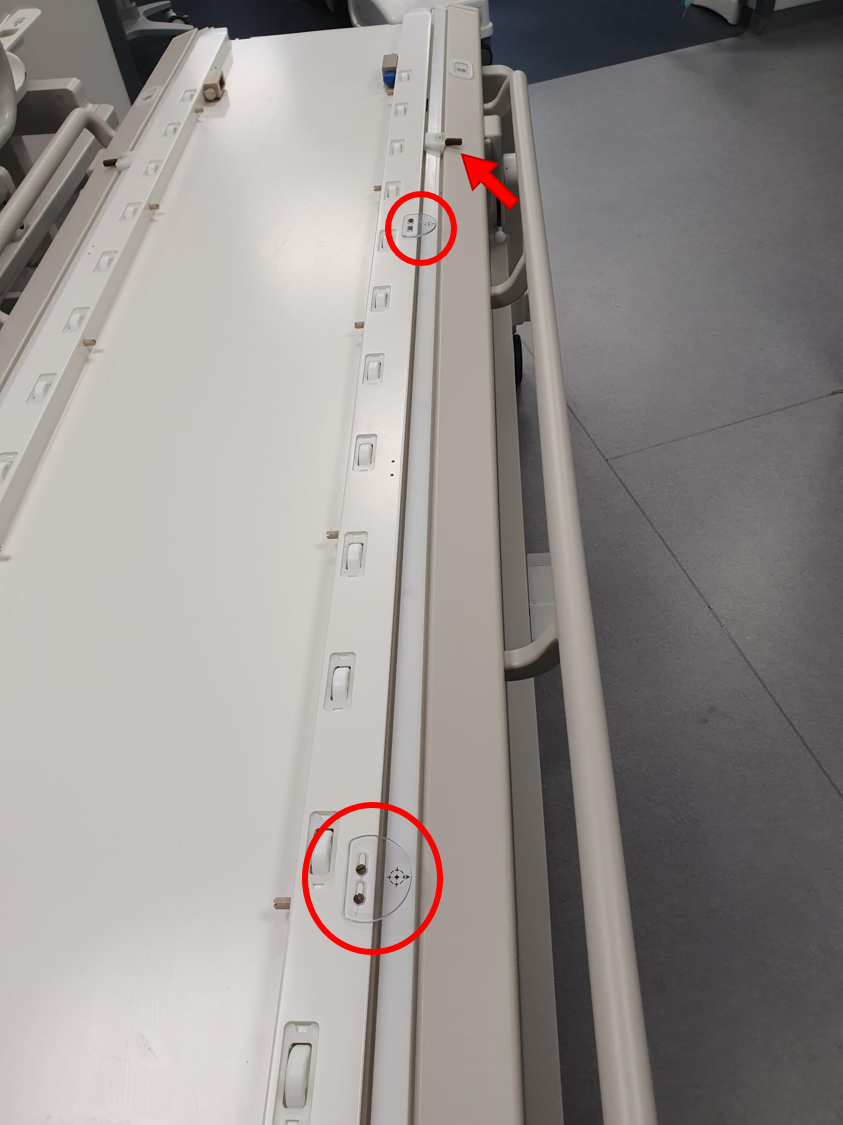


Figure 13 locations on side of manual table of clear plastic markers (circled) and brass screw (indicated with red arrow)

The screw and plastic markers can be used to position the coil in the isocentre by aligning the brass screw with the first marker (as shown in Figure 14) and then moving the bed (see Appendix A for details on how to move table top) into the scanner bore until the second marker is aligned with the screw.


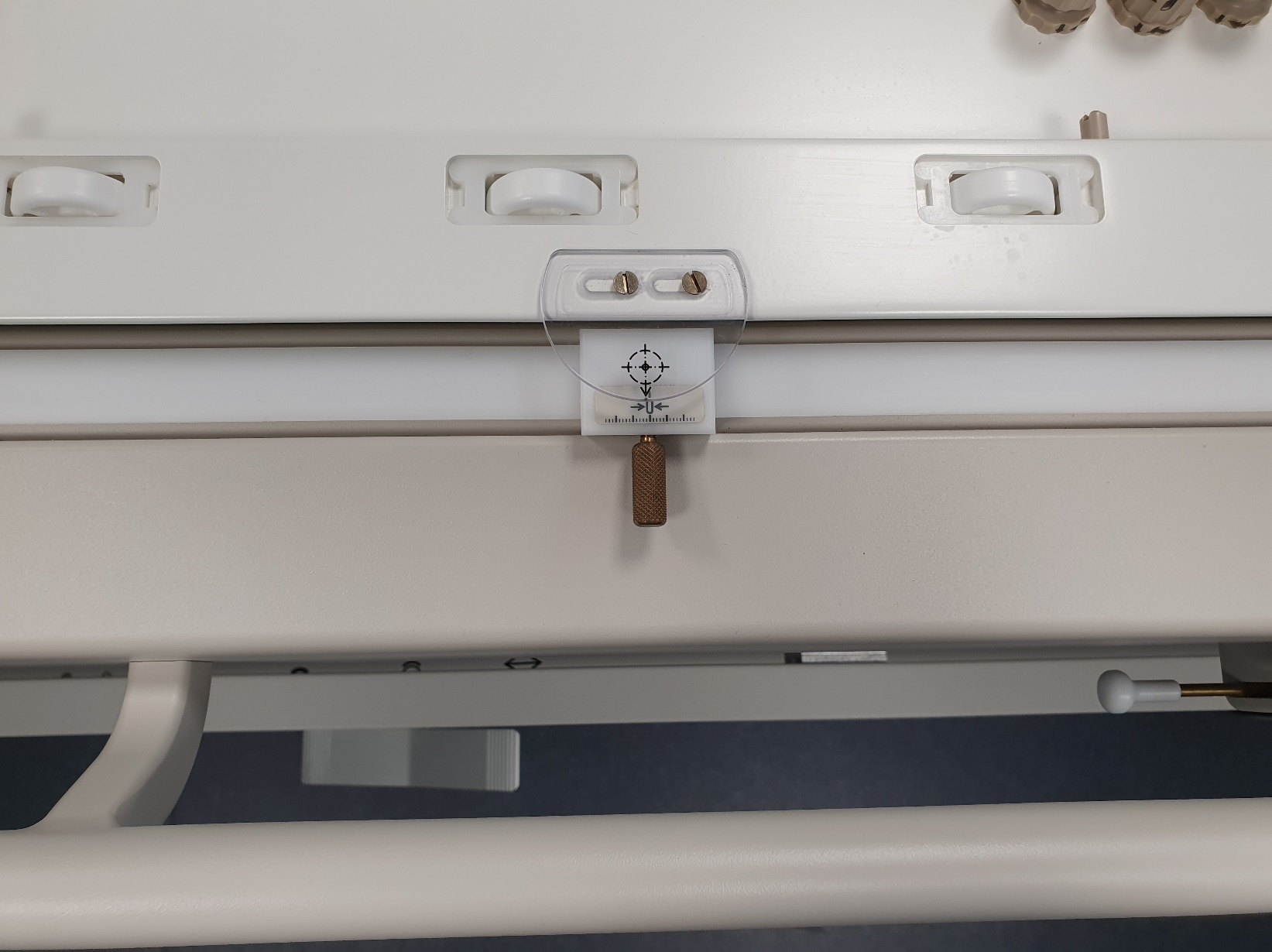


Figure 14 screw and marker for positioning coil in isocentre

**5. ACQUIRING QA IMAGES**

Set up a patient with appropriate patient name and ID (e.g. “NORASQA”, DOB: 01-JAN-1990). Fill in remaining details in a standard manner.

The NORAS QA protocol contains three sequences. The first Survey is a standard 3-plane localiser to determine the location of the phantom in magnet. The second localiser (*T1W_marker_loc*) is a large FOV 3D T1 FFE aiming to capture all 14 fiducials – this localiser is performed during clinical scanning. After acquiring this image the fiducials present in the image should be counted to ensure that all 14 are present. If any are missing then the FOV should be repositioned and the scan rerun.

The final sequence (*T1W_3D_x2*) is a large FOV (400 x 322 x 250 mm) T1 FFE with a 1.5mm isometric voxel size and two dynamics. This should be acquired using the previous localisers to ensure that the entirety of the bottle and fiducial markers are contained in the image and **aligned with the top of the bottle** as shown below. Scan parameters for this sequence are given in the Appendix B.


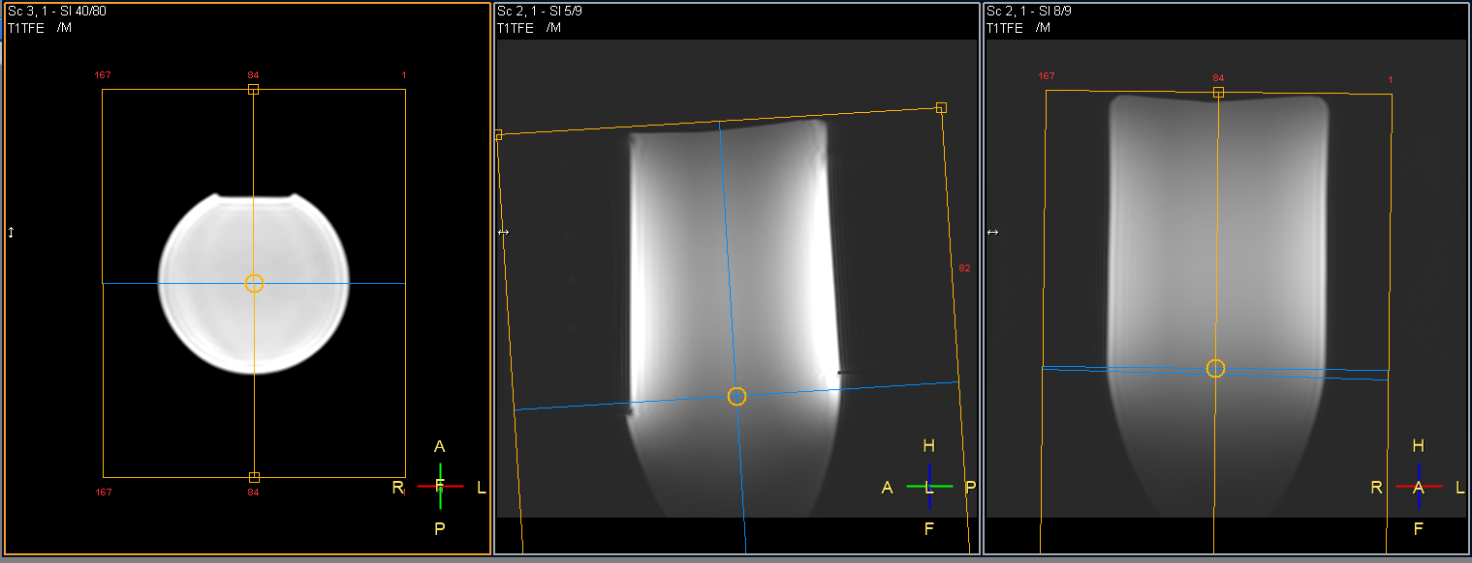


Figure 15 correct positioning for image acquisition

**6. SENDING QA IMAGES**

Once the delated reconstruction is completed, send all images for offline analysis.
